# Alveolar deadspace and intrapulmonary shunt in healthy individuals and in individuals who have recovered from COVID‐19 infection

**DOI:** 10.1113/EP092971

**Published:** 2025-09-04

**Authors:** Dominic Sandhu, Snapper R. M. Magor‐Elliott, Nayia Petousi, Nick P. Talbot, Alexander N. Bennett, David A. Holdsworth, Grant A. D. Ritchie, Peter A. Robbins

**Affiliations:** ^1^ Department of Physiology, Anatomy & Genetics University of Oxford Oxford UK; ^2^ Department of Chemistry University of Oxford Oxford UK; ^3^ Nuffield Department of Medicine University of Oxford Oxford UK; ^4^ Academic Department of Medical Rehabilitation Stanford Hall Loughborough UK; ^5^ Academic Department of Military Medicine Royal Centre of Defence Medicine Birmingham UK

**Keywords:** alveolar–arterial gradients, computed cardiopulmonography, gas exchange, human, ventilation–perfusion mismatch

## Abstract

Following acute COVID‐19 infection, unvaccinated patients have been reported to exhibit elevated alveolar deadspace (*˙V*_D,alv_/*˙V*_T_) and intrapulmonary shunt (*˙Q*_s_/*˙Q*_T_) fractions. However, as there is uncertainty surrounding the upper limits of normal for *˙V*_D,alv_/*˙V*_T_ and *˙Q*_s_/*˙Q*_T_, we sought to replicate the findings from a separate, previously reported cohort of COVID‐19 patients that also included a healthy control group never infected with COVID‐19. Data from 81 participants, classified into four different groups based on the severity of prior COVID‐19 infection, were used. All participants had arterial blood‐gas samples drawn while highly precise measurements of their respiratory gas exchange were made. The gas exchange data were used to estimate alveolar $P_{CO_{2}}$ and $P_{O_{2}}$, and the differences between these values and the corresponding arterial blood‐gas values provided the alveolar–arterial gradients from which *˙V*_D,alv_/*˙V*_T_ and *˙Q*_s_/*˙Q*_T_ were calculated. Mean *˙V*_D,alv_/*˙V*_T_ was 0.115 ± 0.062 and mean *˙Q*_s_/*˙Q*_T_ was 0.014 ± 0.011. No significant differences between the groups, including the uninfected control group, were detected for either *˙V*_D,alv_/*˙V*_T_ or *˙Q*_s_/*˙Q*_T_, although if severity was instead treated as an interval measure, then a small increase in *˙Q*_s_/*˙Q*_T_ with severity (*P* = 0.00934) could be detected. Many participants, including controls, exceeded the originally proposed upper limit of normal for *˙V*_D,alv_/*˙V*_T_, whereas no participant exceeded the originally proposed upper limit for *˙Q*_s_/*˙Q*_T_. We conclude that prior infection with COVID‐19 had no effect on *˙V*_D,alv_/*˙V*_T_ and little effect on *˙Q*_s_/*˙Q*_T_, and that the supposedly high values of *˙V*_D,alv_/*˙V*_T_ are within the normal range.

## Abstract (highlights)

- **What is the central question of this study?**
  Using a three‐compartment lung model to evaluate gas exchange in patients following recovery from COVID‐19 and in uninfected control participants, does prior infection with COVID‐19 affect gas exchange efficiency?
- **What is the main finding and its importance?**
  There was no difference between patients and controls for alveolar deadspace fraction, and any elevation in shunt fraction was minor. Measured values for alveolar deadspace fraction were ∼10‐fold greater than those for intrapulmonary shunt fraction, and this appears to reflect normal physiology rather than any effect of COVID‐19.

## 1 INTRODUCTION

The Riley and Cournand three‐compartment model of the lung (Riley & Cournand, 1949) has provided a powerful theoretical framework for studying gas exchange in the lung. The model is a steady‐state model of gas exchange; it has unidirectional flows for both blood and gas, and there is only one compartment (the ideal compartment) in which gas exchange occurs between blood and gas at the respiratory quotient set by metabolism. The other two compartments comprise an alveolar deadspace compartment, which adds inspired gas to the gas flow coming from the ideal compartment to form the alveolar gas, and a shunt compartment, which adds mixed venous blood to the blood coming from the ideal compartment to form the arterial blood. The enormous power of this simple model is that the effects on alveolar and arterial $P_{CO_{2}}$ and $P_{O_{2}}$ of any real ventilation‐perfusion (*˙V*:*˙Q*) distribution within the lung, no matter how complex, can always be replicated by a three‐compartment model with the relevant gas and blood flows to the three compartments.

Recently, Farrow et al. (2023) estimated the alveolar–arterial differences for $P_{CO_{2}}$ and $P_{O_{2}}$ for patients who had recovered from COVID‐19, and they then effectively used the concepts from the three‐compartment model (Wagner et al., 2022) to estimate the fraction of ventilation flowing to the deadspace compartment (*˙V*_D,alv_/*˙V*_T_) and the fraction of blood flowing to the shunt compartment (*˙Q*_s_/*˙Q*_T_). In this model, *˙V*_D,alv_/*˙V*_T_ reflects both ‘pure’ deadspace (where *˙V*/*˙Q* → ∞) and ‘apparent’ deadspace (generated by regions of high *˙V*/*˙Q*), and similarly *˙Q*_s_/*˙Q*_T_ reflects both ‘pure’ shunt (where *˙V*/*˙Q* = 0) and ‘apparent’ shunt (generated by regions of low *˙V*/*˙Q*). Farrow et al. (2023) found abnormally high values for *˙V*_D,alv_/*˙V*_T_ (overall median value was 16.6%) in 86% of patients that were unrelated to the severity of the prior infection and abnormally high values for *˙Q*_s_/*˙Q*_T_ (overall median value was 4.3%) in 37% of patients that had a partial relationship to the severity of the prior infection. The length of time between infection and measurement (up to 13 months) was not predictive of effect size. As the two effects were poorly correlated with one another, the authors concluded that they were probably associated with two different pathologies.

These findings of Farrow et al. (2023) are striking as they suggest that gas exchange in the lungs of the majority of patients who contracted COVID‐19 without prior vaccination remained abnormal for a long time after the infection had fully resolved. However, a major shortcoming of their approach was that there were no control participants within their main cohort, and there were no prior data using their technique from which the normal ranges had been determined. Instead, the upper limits of normal came from a completely different technique (Wagner et al., 1987) that did not estimate either of these values directly. Furthermore, for seven (younger) healthy participants that they had added to the study to check these limits, three had values for *˙V*_D,alv_/*˙V*_T_ that were either at or above their upper limit of normal and two had values for *˙Q*_s_/*˙Q*_T_ that were close to their upper limit of normal.

The object of the present study was to determine whether the findings of Farrow et al. (2023) could be replicated in a second cohort of patients who had contracted COVID‐19 before vaccines were generally available and in particular using a cohort which included participants who had not contracted COVID‐19. For this purpose, a subset of the MCOVID cohort (Magor‐Elliott et al., 2022; O'Sullivan et al., 2021) was chosen. This group had been studied at ∼6 and/or ∼12 months after the acute infection and importantly contained a set of control participants. All participants had been studied with a molecular flow sensor (Ciaffoni et al., 2016) that provided extremely accurate and highly time‐resolved measures of gas exchange, and most had had an associated timed arterial blood gas sample drawn during the initial air‐breathing phase of the protocol associated with the study.

One difficulty in calculating *˙V*_D,alv_/*˙V*_T_ and *˙Q*_s_/*˙Q*_T_ for the three‐compartment model is that values for the alveolar $P_{CO_{2}}$ and $P_{O_{2}}$ generally have to be estimated from the respiratory data. Farrow et al. (2023) approached this problem by regressing airway $P_{CO_{2}}$ and $P_{O_{2}}$ values against exhaled volume for the alveolar plateau (phase 3) phase of exhalation and then taking a midpoint value. In the present study, we used two approaches, both of which made use of a large, tidally ventilated model of the cardiopulmonary system that had been used in the previous study (Magor‐Elliott et al., 2022). In the first approach, the model was fitted individually to each data set, and the alveolar $P_{CO_{2}}$ and $P_{O_{2}}$ were estimated directly from the simulated data. In the second approach, the alveolar $P_{CO_{2}}$ and $P_{O_{2}}$ values were not estimated directly, and instead, alveolar deadspace was introduced as a feature of the model and adjusted in size until, on a $P_{CO_{2}}$–$P_{O_{2}}$ diagram, the blood *R*‐line of the model passed through the measured arterial blood gas point. These two approaches are explained in more detail in Section 2.

## 2 METHODS

### 2.1 Ethical approval

Ethical approval was received from the Ministry of Defence Research Ethics Committee (1061/MODREC/20). Except for public registration, the study conformed to all the principles of the *Declaration of Helsinki* of 2013, with all participants providing informed consent.

### 2.2 MCOVID cohort

The MCOVID cohort was formed from British military personnel who had contracted COVID either in 2020 or early in 2021 before any of the participants had received a vaccination (Magor‐Elliott et al., 2022). The cohort also included a number of control participants who were recruited through poster adverts at military units. These participants were excluded if they had any symptoms suggestive of COVID infection after February 2020. They all underwent both PCR COVID‐19 surface antigen testing and COVID‐19 antibody testing and were excluded if either test was positive. The participants were split into four groups based on the severity of the original infection: (1) Control, where the participant was uninfected (World Health Authority (WHO) severity scale (Marshall et al., 2020) of 0); (2) Community, where the participant was managed in the community (WHO scale 1–3); (3) Ward, where the patient was managed in hospital with ventilatory support including O_2_ therapy, but was not mechanically ventilated (WHO scale 4–6); and (4) ICU, where the patient was managed in an intensive care unit (ICU) using invasive mechanical ventilation or, rarely, extracorporeal membrane oxygenation (WHO scale 7–9). Participants were studied at a median of 22 weeks after they had recovered from the acute infection, and a subset of participants undertook a second study visit ∼6 months later.

### 2.3 Protocol

The experimental work undertaken on this cohort has already been described in a previous report (Magor‐Elliott et al., 2022). Briefly, during each visit, the participant would breathe through a mouthpiece with their nose occluded for ∼12 min. For the first ∼7 min, the participant breathed air, and for the following ∼5 min, the participant breathed pure O_2_ to wash the N_2_ out of the lungs. During the 12 min protocol, the respiratory flow and respired gas compositions were logged every 10 ms with a bespoke, highly precise, molecular flow sensor (Ciaffoni et al., 2016) for later analysis. Most participants also gave consent for an arterial blood sample to be drawn from their radial artery during the course of the air‐breathing period of the protocol. In these participants, the region surrounding the radial artery was infiltrated with local anaesthetic prior to undertaking the protocol on the mouthpiece to ensure that the sample could be drawn with the minimum of disturbance to the participant.

### 2.4 Data analysis

#### 2.4.1 Model fitting and estimation of alveolar $P_{CO_{2}}$ ($P_{\mathrm{AC}O_{2}}$) and $P_{O_{2}}$ ($P_{AO_{2}}$)

The process of fitting the cardiopulmonary model to the data and the parameter values retrieved from so doing has already been described in the previous report (Magor‐Elliott et al., 2022). Essentially, the ‘breathing’ of the model was driven by the measured respiratory flows, and the model was supplied with the measured inspiratory gas compositions. The parameters of the model then determine the profiles for the expiratory gas composition. If the parameters of the model do not reflect the participant's physiology, then the expiratory gas concentration profiles from the model will not match those from the participant. In order to identify the specific parameter values associated with each participant, the cardiopulmonary model was incorporated within a non‐linear least squares optimisation algorithm that progressively adjusted the parameters of the model so as to minimise the deviation between the expiratory gas profiles from the model and those from the participant. More detail is given in the previous report (Magor‐Elliott et al., 2022).

**FIGURE 1:**
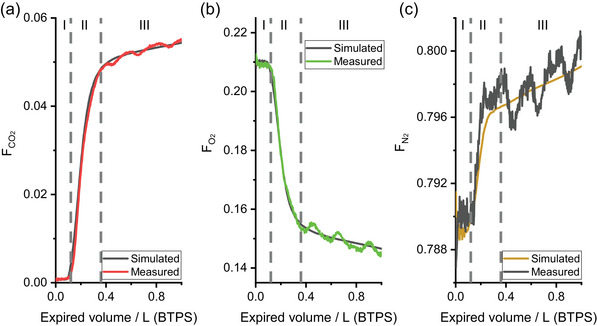
Detail illustrating fit of model to data for an example single breath. (a) Expired CO_2_ fraction ($F_{CO_{2}}$) versus expired volume. (b) Expired O_2_ fraction ($F_{O_{2}}$) versus expired volume. (c) Expired N_2_ fraction versus expired volume ($F_{N_{2}}$). The oscillations in phase III of the expired fractions for the measured data are cardiogenic in origin.

Figure 1 illustrates the expiratory concentration profiles associated with one participant after the fit of the model. Phase I (exhalation of purely deadspace gas), phase II (exhalation of a mixture of deadspace gas and alveolar gas) and phase III (exhalation of purely alveolar gas) are clearly visible in the profiles for CO_2_, O_2_ and N_2_ for both model and data. The modelling process uses the whole of the expiratory gas profile, and it forms the total expired volume from the fraction that is associated with anatomical and apparatus deadspace and the fraction that is associated with alveolar ventilation.

In the present study, the model was rerun on the datasets after the software had been modified to report a collection of averages from the model for a set of overlapping time periods of ∼30 s before the arterial sample was drawn. The precise length of each of these time periods was adjusted so that the averages were generated from model data for an integer number of breaths (i.e., no part breaths). These averages included:

1. The rate of CO_2_ and O_2_ exchange (${\dot{V}}_{CO_{2}}$ and ${\dot{V}}_{O_{2}}$) between the blood and gas in the model alveolar compartments, summed across all the pulmonary blood vessels of the model,
2. The flow‐weighted sum of the CO_2_ and O_2_ partial pressures of the gas entering the alveolar lung units during inspiration to provide averages for ‘mixed‐inspired’ $P_{CO_{2}}$ and $P_{O_{2}}$ ($P_{I,\mathrm{mixC}O_{2}}$ and $P_{I,\mathrm{mix}O_{2}}$),
3. The flow‐weighted sum of the CO_2_ and O_2_ partial pressures of all the gas leaving the alveolar lung units during expiration to provide averages for $P_{\mathrm{AC}O_{2}}$ and $P_{AO_{2}}$.

#### 2.4.2 Calculation of *˙V*_D,alv_/*˙V*_T_ and *˙Q*_s_/*˙Q*_T_

**FIGURE 2:**
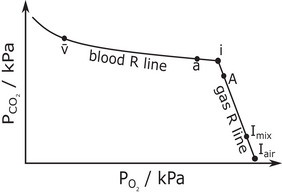
Calculation of the ideal point. (1) Plot the arterial (a) and alveolar (A) points on the $P_{CO_{2}}$–$P_{O_{2}}$ diagram. The alveolar point is calculated from the total gas exiting the gas exchanging units of the model for the ∼30 s period immediately prior to the arterial blood gas sample. (2) Obtain the respiratory exchange ratio (*R*) for the gas exchange between blood and alveolus for the same ∼30 s period immediately prior to the arterial sample. (3) Calculate gas *R*‐line through the alveolar point and, using the patient's particular blood gas values, the blood *R*‐line through the arterial point. (4) Identify the ideal (*i*) point at the intersection of the two lines. Also shown are the mixed venous point (*v̄*), identified from the model cardiac output and the CO_2_ and O_2_ exchange rates for ∼30 s period immediately prior to the arterial blood gas sample; and two inspired points, one for air (*I*_air_) and the other for the total mixed inspired gas (*I*_mix_) entering the alveoli for the same ∼30 s period.

The values for ${\dot{V}}_{CO_{2}}$ and ${\dot{V}}_{O_{2}}$ from the ∼30 s period immediately prior to the arterial sampling gave a value for the respiratory exchange ratio, *R*, associated with the blood sample. This allowed a blood *R*‐line to be constructed through the arterial $P_{CO_{2}}$ and $P_{O_{2}}$ ($P_{\mathrm{aC}O_{2}}$ and $P_{aO_{2}}$) on a $P_{CO_{2}}$–$P_{O_{2}}$ diagram using a physicochemical model of CO_2_ and O_2_ carriage by blood (O'Neill & Robbins, 2017), as illustrated in Figure 2. This blood model was constructed using each participant's own values for haemoglobin and other variables from the blood gas analysis. Also plotted on the $P_{CO_{2}}$–$P_{O_{2}}$ diagram were the $P_{\mathrm{AC}O_{2}}$ and $P_{AO_{2}}$ values determined during the 30 s period prior to the blood gas sample being drawn. A gas *R*‐line through this point was constructed using the alveolar gas equation and the same *R*‐value. The intersection of these two lines identifies the ideal $P_{CO_{2}}$ and $P_{O_{2}}$ ($P_{\mathrm{iC}O_{2}}$ and $P_{iO_{2}}$), as described by Riley and Cournand (Riley & Cournand, 1949).

The magnitude of the intrapulmonary shunt is reflected by the difference between $P_{iO_{2}}$ and $P_{aO_{2}}$ (and similarly between $P_{\mathrm{aC}O_{2}}$ and $P_{\mathrm{iC}O_{2}}$). To calculate a value for *˙Q*_s_/*˙Q*_T_, the relationships:

$${\dot{V}}_{CO_{2}}={\dot{Q}}_{T}\left(C_{\bar{v}CO_{2}}-C_{\mathrm{aC}O_{2}}\right)$$

and

$${\dot{V}}_{O_{2}}={\dot{Q}}_{T}\left(C_{aO_{2}}-C_{\bar{v}O_{2}}\right)$$

were used to determine the mixed venous CO_2_ and O_2_ contents, $C_{\bar{v}CO_{2}}$ and $C_{\bar{v}O_{2}}$, where ${\dot{V}}_{CO_{2}}$ and ${\dot{V}}_{O_{2}}$ were the values from the 30 s period prior to the arterial sampling, $C_{\mathrm{aC}O_{2}}$ and $C_{aO_{2}}$ were the arterial CO_2_ and O_2_ contents calculated from the blood gas measurement and *˙Q*_T_ was the cardiac output employed in the model (based on metabolism).

*˙Q*_s_/*˙Q*_T_ is then given by either

$${\dot{Q}}_{s}/{\dot{Q}}_{T}=\left(C_{\mathrm{aC}O_{2}}-C_{\mathrm{iC}O_{2}}\right)/\left(C_{\bar{v}CO_{2}}-C_{\mathrm{iC}O_{2}}\right)$$

or

$${\dot{Q}}_{s}/{\dot{Q}}_{T}=\left(C_{iO_{2}}-C_{aO_{2}}\right)/\left(C_{iO_{2}}-C_{\bar{v}O_{2}}\right)$$

where $C_{\mathrm{iC}O_{2}}$ and $C_{iO_{2}}$ are the CO_2_ and O_2_ contents associated with the ideal point.

A value for *˙V*_D,alv_/*˙V*_T_ can be calculated as either


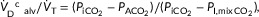

or


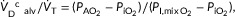

where the superscript ‘c’ indicates that these particular values for *˙V*_D,alv_/*˙V*_T_ have been calculated using estimates for alveolar gas composition. (Note that because we define the alveolar gas composition as the average composition of all the gas leaving the alveoli during expiration, then the corresponding inspired gas composition is that of all the gas entering the alveoli during inspiration, including that rebreathed from the anatomical/apparatus deadspace. If fresh air had instead been used as the inspired gas composition, then the corresponding alveolar gas composition would need to be just that of the alveolar gas that both leaves the alveoli and also exits the anatomical/apparatus deadspace. The two approaches will necessarily give different values for the inspired and alveolar gas compositions, but both should result in the same alveolar deadspace fraction.)

#### 2.4.3 Estimation of *˙V*_D,alv_/*˙V*_T_ using an explicit model of alveolar deadspace

As an alternative to using values for $P_{\mathrm{AC}O_{2}}$ and $P_{AO_{2}}$ to calculate *˙V*_D,alv_/*˙V*_T_, an explicit model of alveolar deadspace can be formed by replicating the 125 compartments of the standard cardiopulmonary model with a further scaled set of 125 compartments that match the underlying properties of the original 125 compartments, except that they have no blood flow. The alveolar deadspace can then be modelled by diverting a fraction of the inspired gas flow away from the standard compartments and into the unperfused compartments. In this model, the total end‐expiratory alveolar volume and total anatomical deadspace are partitioned in the same ratio as for the gas flow between the perfused and unperfused sets of compartments. If for any dataset the measured arterial point fell on or below the model *R*‐line, then *˙V*_D,alv_/*˙V*_T_, was set equal to zero.

Alveolar deadspace can arise both as ‘pure’ alveolar deadspace, as modelled above and as ‘apparent’ alveolar deadspace that arises from regions with high *˙V*/*˙Q* ratios in the lung. Thus, in order to identify all the alveolar deadspace using a model of pure alveolar deadspace, it was necessary to minimise any *˙V*:*˙Q* inequalities arising from the 125 model alveolar units that are perfused. This was achieved by adjusting the parameters of the model as described in the next section.

Once the adjustments had been made, the model was first fit with no alveolar deadspace ventilation. The model was then repeatedly refit, adjusting the model alveolar deadspace fraction (*˙V*_D_^m^_alv_/*˙V*_T_) until the blood *R*‐line associated with the model lung for the 30 s of blood flow immediately prior to the arterial sample passed through the measured blood‐gas values for $P_{\mathrm{aC}O_{2}}$ and $P_{aO_{2}}$. The superscript ‘m’ denotes that these values for *˙V*_D,alv_/*˙V*_T_ were obtained by introducing a model of the alveolar deadspace directly into the large cardiopulmonary model.

#### 2.4.4 Predicted $P_{\mathrm{aC}O_{2}}$ and $P_{aO_{2}}$ from model with varying $\dot{V}\;:\;\dot{Q}$ inequality

Four parameters of the cardiopulmonary model interact to generate *˙V*:*˙Q* inequality. These are the standard deviation of the standardised deadspace (σ*V* d) and the three parameters that specify the form of the bivariate log‐normal distribution of lung compliance and conductance. The distribution is defined by the standard deviation of the logarithm of the standardised lung compliance (σln*C* l); the standard deviation of the logarithm of the standardised vascular conductance (σln*C*d); and the correlation coefficient between these two variables (ρ). σ*V* d and σln*C* l play major roles in shaping the expired gas profile and necessarily have to be estimated separately for each participant. Although they have a major influence on the efficiency of gas exchange, the influence of σln*C*d and ρ on the expired gas profile is very minor and limited to second‐order effects. The corollary is that σln*C*d and ρ can only be poorly estimated, if at all, from fitting the model to the expired gas profile.

The standard practice we have adopted is to fix σln*C*d to be 0.3 greater than σln*C* l and set ρ equal to 0.87. These values give rise to a *˙V*:*˙Q* distribution that is symmetric and similar to that reported for healthy controls (Wagner et al., 1974). However, to estimate alveolar deadspace explicitly (as described in the previous section), it is necessary to minimise the contribution from *˙V*:*˙Q* inequality in the model. To achieve this, σln*C*d was fixed to equal σln*C* l and ρ was set very close to 1 (for reasons relating to the code, a value of 0.98 was used). In order to explore how wider *˙V*:*˙Q* distributions reduce the efficiency of gas exchange and so increase the model $P_{\mathrm{aC}O_{2}}$ and decrease the model $P_{aO_{2}}$, σln*C*d can be increased and/or ρ reduced. Here, we explored fixing σln*C*d to be 0.45 above σln*C* l and separately also reducing ρ to 0.7.

The cardiopulmonary model was fit to each participant for each of the four pairs of values for σln*C*d and ρ above, and from these fits, four pairs of model values for $P_{\mathrm{aC}O_{2}}$ and $P_{aO_{2}}$ (notated as a_1_, a_2_, a_3_ and a_4_, in ascending order of *˙V*:*˙Q* mismatch) were determined. In all cases, the *˙V*:*˙Q* distributions were effectively symmetric because they were all generated via a bivariate log‐normal distribution. For all four fits, $P_{\mathrm{AC}O_{2}}$ and $P_{AO_{2}}$ will be essentially identical because they are effectively determined by the expired gas profiles for $P_{CO_{2}}$ and $P_{O_{2}}$, respectively.

#### 2.4.5 Statistical analysis

Statistical analysis was undertaken using IBM SPSS Statistics v29.0 (IBM Corp., Armonk, NY, USA) and was conducted using general linear models. The COVID‐19 severity group was generally incorporated into the model as a fixed factor, although where there appeared to be a progressively increasing effect with severity, it was also studied as an interval measure (i.e., as a covariate). Statistical significance was assumed at *P *< 0.05.

### 2.5 Quality control

The experimental work underlying this study was conducted during the course of a pandemic in a clinical environment on participants who were generally naïve to such studies by a number of different individuals. For this reason, we reviewed each blood gas data point, each respiratory protocol and the stability of each participant's breathing in the period associated with the blood gas sample to determine whether certain datasets should be excluded. Blood gas results were checked to ensure that they were not obviously venous rather than arterial in nature, and also whether any had implausibly high values for $P_{aO_{2}}$, such as can arise through an air bubble in the sample. The nitrogen balance during the air‐breathing period of the protocol was checked to ensure the absolute value was <60 mL/min. Values above 60 mL/min tend to be associated with imperfect seals around the mouthpiece or noseclip, and so any studies associated with such values were rejected. Finally, it is not possible to time align an arterial sample drawn from the radial artery precisely with an exact time point in the lung that generated the sample. Thus, in order to avoid significant errors, it is important that the gas exchange is reasonably stable in the lung over the period associated with the arterial sample. This was checked using the model values for $P_{\mathrm{AC}O_{2}}$ and $P_{AO_{2}}$ for the 30 s averages over the 90 s period immediately prior to the arterial sample. If the coefficient of variation for either $P_{\mathrm{AC}O_{2}}$ or $P_{AO_{2}}$ exceeded 5%, the study was rejected.

## 3 RESULTS

### 3.1 Participants

In total, there were 129 studies in which arterial blood sampling was performed. Eight of the samples were judged unreliable. A further eight studies were rejected because the measured nitrogen balance exceeded 60 mL/min. The model failed to fit the data for two of the studies and finally, an additional five studies were rejected after the modelling had been completed (see below) because the coefficient of variation for $P_{\mathrm{AC}O_{2}}$ or $P_{AO_{2}}$ exceeded 5% during the 90 s period immediately prior to arterial blood gas sampling. This left 106 studies in 81 different participants, with 25 participants providing satisfactory data for both the initial and the follow‐up visit. The final number of participants in each clinical group together with their physical characteristics are reported in Table 1.

**TABLE 1:** Participant characteristics.

	COVID severity group				Overall
	Control	Community	Ward	ICU	
Number of participants	15	41	20	5	81
Female (*n* (%))	3 (20)	4 (10)	3 (20)	0 (0)	10 (12)
Age (years)	38.5 ± 12.4	36.8 ± 10.4	40.8 ± 11.3	37.8 ± 20.0	39.5 ± 10.1
Height (m)	1.65 ± 0.43	1.76 ± 0.29	1.68 ± 0.38	1.48 ± 0.66	1.76 ± 0.21
Weight (kg)	74.6 ± 20.8	90.4 ± 22.6	90.5 ± 23.1	81.5 ± 39.4	90.0 ± 18.7
BMI (kg/m^2^)	24.1 ± 6.6	27.6 ± 5.6	29.0 ± 7.5	26.0 ± 12.6	28.2 ± 5.0
Smokers (*n* (%))	0	1 (2)	0	1 (20)	2 (3)
Ex‐smokers (*n* (%))	1 (7)	3 (7)	7 (35)	3 (60)	14 (17)
Asthma (*n* (%))	0	1 (2)	1 (5)	1 (20)	3 (4)
COPD (*n*)	0	0	0	0	0
Diabetes (*n* (%))	0	1 (2)	1 (5)	0	2 (3)
CHD (*n*)	0	0	0	0	0
Hypertension (*n* (%))	0	3 (7)	1 (5)	0	4 (5)

*Note*: Values are means ± SD. Abbreviations: BMI, body mass index; COPD, chronic obstructive pulmonary disease; CHD, chronic heart disease.

### 3.2 Analysis of measured $P_{\mathrm{aC}O_{2}}$ and $P_{aO_{2}}$

ANOVA was first performed on the subset of 25 participants with repeat studies. No significant effect of visit was found, and therefore, the data from both visits were pooled across all the participants, with the values averaged for the participants who had two data sets.

**FIGURE 3:**
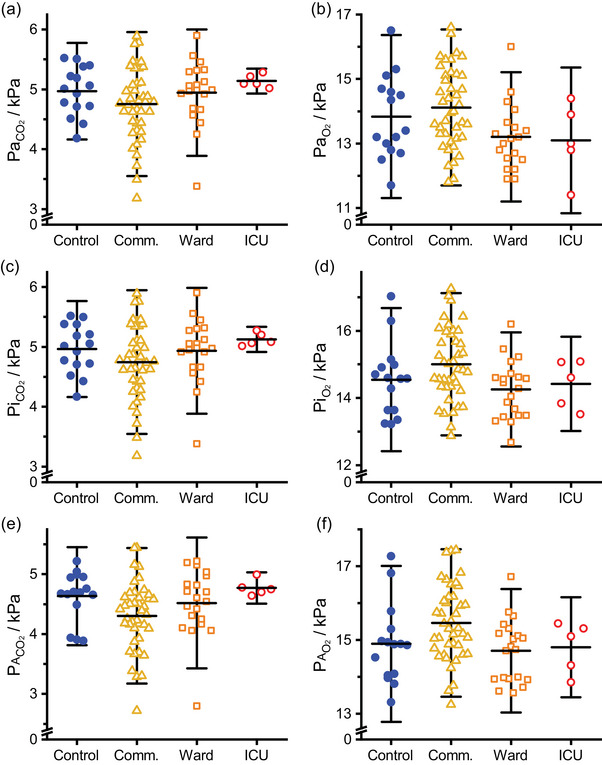
Arterial, ideal and alveolar partial pressures for CO_2_ and O_2_ for different COVID‐19 severity groups. (a, b) Arterial $P_{CO_{2}}$ ($P_{\mathrm{aC}O_{2}}$) and $P_{O_{2}}$ ($P_{aO_{2}}$). (c, d) Ideal $P_{CO_{2}}$ ($P_{\mathrm{iC}O_{2}}$) and $P_{O_{2}}$ ($P_{iO_{2}}$). (e, f) Alveolar $P_{CO_{2}}$ ($P_{\mathrm{AC}O_{2}}$) and $P_{O_{2}}$ ($P_{AO_{2}}$). Horizontal bars indicate mean values, whiskers show ±1.96 standard deviations. *n* = 15, 41, 20, 5 for the control, community (comm.), ward and intensive care unit (ICU) groups, respectively.

After pooling, a linear model was used to determine whether the results from any of the patient groups differed from the control group. No significant effects were found. Mean values and standard deviation (SD) were: $P_{\mathrm{aC}O_{2}}$ 4.86 ± 0.55 kPa and $P_{aO_{2}}$ 13.77 ± 1.24 kPa (*n* = 81 in both cases). The data are illustrated in Figure 3a,b.

### 3.3 Visual check on quality of model fit

**FIGURE 4:**
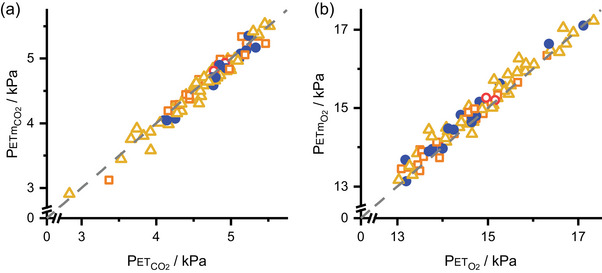
Comparison of model predicted and measured end‐tidal $P_{CO_{2}}$ and $P_{O_{2}}$. (a) Model end‐tidal $P_{CO_{2}}$ ($P_{\mathrm{ETmC}O_{2}}$) plotted against measured end‐tidal $P_{CO_{2}}$ ($P_{\mathrm{ETC}O_{2}}$). (b) Model end‐tidal $P_{O_{2}}$ ($P_{\mathrm{ETm}O_{2}}$) plotted against measured end‐tidal $P_{O_{2}}$ ($P_{\mathrm{ET}O_{2}}$). Broken line, line of identity.

Visual checks were undertaken to confirm that the expiratory gas profiles from the model fitted the data well. By way of a summary across all studies, Figure 4 compares the predicted end‐tidal gas compositions for CO_2_ and O_2_, which should lie on the end‐tidal plateau, with the measured end‐tidal values for CO_2_ and O_2_. The agreement between the paired values is good.

### 3.4 Ideal and alveolar gas values, shunt and alveolar deadspace

The calculated ideal and alveolar gas values are shown in Figure 3c–f. With one exception, a linear model did not detect any significant difference between the control group and any of the groups that had been infected with COVID‐19. The exception was for $P_{\mathrm{AC}O_{2}}$, where the value was 0.33 kPa lower in the community group (*n* = 41) compared with the control group (*n* = 15). The group means were 4.30 kPa and 4.63 kPa, respectively; the mean difference was 0.33 kPa with a confidence interval of 0.001–0.650 kPa, in Student's two‐sample *t*‐test *P* = 0.0482). Overall mean values and SD were: $P_{\mathrm{iC}O_{2}}$ 4.86 ± 0.55 kPa, $P_{iO_{2}}$ 14.70 ± 1.05 kPa, $P_{\mathrm{AC}O_{2}}$ 4.45 ± 0.55 kPa and $P_{AO_{2}}$ 15.13 ± 1.02 kPa (*n* = 81 in all cases).

**FIGURE 5:**
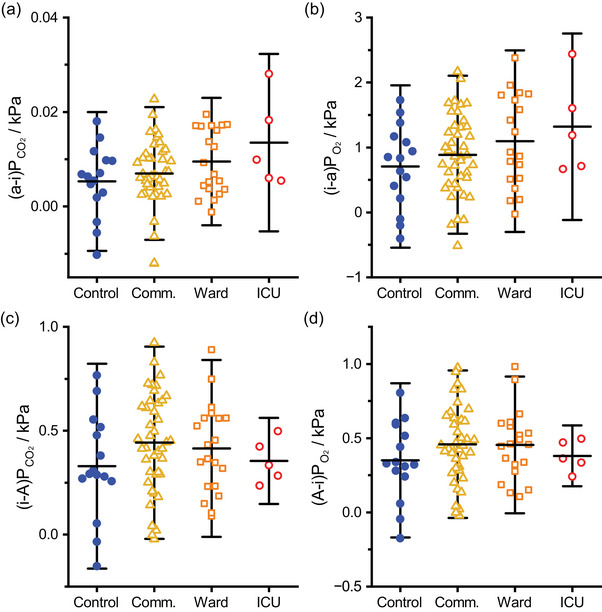
Ideal‐arterial and ideal‐alveolar partial pressure gradients for CO_2_ and O_2_ for different COVID‐19 severity groups. (a, b) Arterial–ideal gradient for CO_2_ ((a‐i)$P_{CO_{2}}$) and ideal–arterial gradient for O_2_ ((i‐a)$P_{O_{2}}$). (c, d) Ideal–alveolar gradient for CO_2_ ((i‐A)$P_{CO_{2}}$) and alveolar–ideal gradient for O_2_ ((A‐i)$P_{O_{2}}$). Horizontal bars indicate mean values, whiskers show ±1.96 standard deviations. *n* = 15, 41, 20, 5 for the control, community (comm.), ward and intensive care unit (ICU) groups, respectively.

The differences between the ideal and arterial points and the ideal and alveolar points are shown in Figure 5a–d. For CO_2_, the differences were much smaller for the arterial‐to‐ideal (a‐i) gradient than for the ideal‐to‐alveolar (i‐A) gradient. Overall, there were no significant differences between the groups. Mean values and SD were: (a‐i)$P_{CO_{2}}$ 0.008 ± 0.007 kPa, (i‐a)$P_{O_{2}}$ 0.92 ± 0.66 kPa, (i‐A)$P_{CO_{2}}$ 0.41 ± 0.23 kPa and (A‐i)$P_{O_{2}}$ 0.43 ± 0.24 kPa (*n* = 81 in all cases). Although none of the groups was significantly different from control, there was a clear suggestion that the (a‐i)$P_{CO_{2}}$ and (i‐a)$P_{O_{2}}$ values increased with increasing COVID‐19 severity and if severity were treated as an interval measure (i.e., as a covariate in the general linear model), then these magnitudes were found to increase with increasing severity (*P* = 0.0150 and *P* = 0.0352, respectively).

**FIGURE 6:**
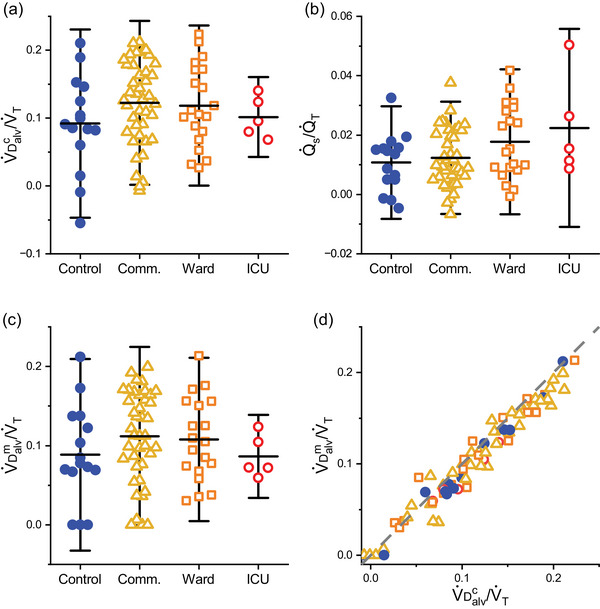
Alveolar deadspace and shunt fractions for different severity groups. (a) Calculated alveolar deadspace fraction (*˙V*_D_^c^_alv_/*˙V*_T_). (b) Calculated intrapulmonary shunt fraction (*˙Q*_s_/*˙Q*_T_). (c) Model‐based estimate of alveolar shunt fraction (*˙V*_D_^m^_alv_/*˙V*_T_). (d) Plot of *˙V*_D_^m^_alv_/*˙V*_T_ against *˙V*_D_^c^_alv_/*˙V*_T_. Broken line, line of identity. Horizontal bars indicate mean values, whiskers show ±1.96 standard deviations. *n* = 15, 41, 20, 5 for the control, community (comm.), ward and intensive care unit (ICU) groups, respectively.

The values for *˙V*_D_^c^_alv_/*˙V*_T_ and *˙Q*_s_/*˙Q*_T_ are shown in Figure 6a,b. Overall, no significant differences were detected between the groups. Mean values and SD were: *˙V*_D_^c^_alv_/*˙V*_T_ 0.115 ± 0.062 and *˙Q*_s_/*˙Q*_T_ 0.014 ± 0.011 (*n* = 81 in both cases). However, as for the (a‐i)$P_{CO_{2}}$ and (i‐a)$P_{O_{2}}$ differences, values for *˙Q*_s_/*˙Q*_T_ appeared to increase with increasing COVID‐19 severity. If severity were treated as an interval measure (i.e., as a covariate in the general linear model), then *˙Q*_s_/*˙Q*_T_ increased with increasing severity (*P* = 0.00934). Finally, the analyses were repeated to include age as a covariate. No significant effects of age were detected.

### 3.5 Estimation of *˙V*_D,alv_/*˙V*_T_ using an explicit model of alveolar deadspace

This method of calculating deadspace succeeded in 80 out of 81 participants. For one of the 106 datasets, the fitting routine failed to run to completion. The differences in $P_{\mathrm{AC}O_{2}}$ and $P_{AO_{2}}$ between the standard model and the new model with the extra deadspace compartments and near‐perfect *˙V*:*˙Q* matching in the perfused compartments were tiny (for CO_2_ this difference was 0.018 ± 0.017 kPa and for O_2_ this difference was 0.001 ± 0.022 kPa, *n* = 80 in both cases).

Figure 6c illustrates *˙V*_D_^m^_alv_/*˙V*_T_ by participant group. No significant differences were detected between the groups. The mean and SD for *˙V*_D_^m^_alv_/*˙V*_T_ was 0.105 ± 0.056 (*n* = 80). Figure 6d is a plot of *˙V*_D_^m^_alv_/*˙V*_T_ against *˙V*_D_^c^_alv_/*˙V*_T_ to provide a comparison between the two different approaches used to estimate *˙V*_D,alv_/*˙V*_T_. For almost all participants, the agreement between the two approaches for estimating *˙V*_D,alv_/*˙V*_T_ was very good.

### 3.6 Locus for predicted arterial points for symmetric *˙V*:*˙Q* distributions

**FIGURE 7:**
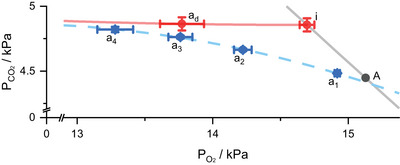
$P_{CO_{2}}$–$P_{O_{2}}$ diagram comparing measured $P_{\mathrm{aC}O_{2}}$ and $P_{aO_{2}}$ data with pairs of predicted values for $P_{\mathrm{aC}O_{2}}$ and $P_{aO_{2}}$ assuming symmetric ventilation–perfusion distributions of differing widths. A, mean alveolar point; a_d_, mean arterial point for the arterial blood gas data; i, mean ideal point; a_1_, a_2_, a_3_ and a_4_, model arterial points for a symmetric ventilation–perfusion distribution of increasing width. Note that the locus of these points passes beneath a_d_, indicating that a symmetric ventilation–perfusion distribution cannot by itself explain both the observed (a‐i) and (i‐A) gradients. Error bars reflect the differences relative to the alveolar values for the participants and show ±2 SE.

Figure 7 is a $P_{CO_{2}}$–$P_{O_{2}}$ diagram on which the mean data for the arterial blood gas values, the mean ideal point and the mean alveolar gas point are illustrated together with the mean blood and gas *R*‐lines. Also shown on this plot are a number of predicted arterial points from the model for different widths for the *˙V*:*˙Q* distribution, with the narrowest at a_1_ and the widest at a_4_. This locus does not pass through the section of the blood *R*‐line that lies between the measured arterial value and the ideal point, illustrating that a symmetric *˙V*:*˙Q* distribution cannot by itself generate such a major disparity between the magnitudes for *˙V*_D,alv_/*˙V*_T_ (0.115) and *˙Q*_s_/*˙Q*_T_ (0.014).

## 4 DISCUSSION

This study found no significant differences between the values for *˙V*_D,alv_/*˙V*_T_ for the patients who had recovered from COVID‐19 and the values for the healthy control group. It therefore does not support the conclusion drawn by Farrow et al. (2023) that *˙V*_D,alv_/*˙V*_T_ is significantly increased in 86% of patients following recovery from COVID. The values for *˙Q*_s_/*˙Q*_T_ were an order of magnitude smaller than those for *˙V*_D,alv_/*˙V*_T_ and in keeping with the commonly accepted view that *˙Q*_s_/*˙Q*_T_ is small in healthy individuals (Roca & Wagner, 1994). However, for *˙Q*_s_/*˙Q*_T_ there did appear to be a modest increase in value with increasing severity of the prior COVID‐19 infection. With the exception of just one or two patients, however, the values could not be considered clinically significant.

While there may be no directly comparable values for *˙V*_D,alv_/*˙V*_T_ that have been derived from CO_2_ published in the literature, it is nevertheless possible to estimate a value from previously published literature. First, there are multiple publications that provide measures of the end‐tidal to arterial (ET‐a) $P_{CO_{2}}$ gradient (Bjurstedt et al., 1962; Matell, 1963; Wasserman et al., 1967; Whipp & Wasserman, 1969). Whipp and Wasserman (1969) summarise the position by stating that the literature is consistent in estimating the ET‐a gradient at ∼−2.5 mmHg (−0.33 kPa) at rest. To estimate the A‐a gradient, a value for the alveolar to end‐tidal (A‐ET) gradient then has to be added to the ET‐a gradient. The A‐ET gradient is caused by the oscillation in alveolar $P_{CO_{2}}$ generated by tidal breathing (DuBois et al., 1952). In simple terms, the end‐tidal value is the peak value for the alveolar oscillation, and the alveolar value is the mean value for the alveolar oscillation. While the amplitude of these oscillations will vary between subjects, a reasonable peak‐to‐trough value is ∼3.8 mmHg (0.51 kPa) (calculated from a CO_2_ production rate of 250 mL/min entering into the effective lung volume of 2.5 L over the course of an expiration lasting 3 s). The corresponding mean‐to‐peak (A‐ET gradient) is half this at ∼−1.9 mmHg (−0.25 kPa). The A‐a gradient is obtained by adding the A‐ET and the ET‐a gradient and is therefore ∼−4.4 mmHg (−0.58 kPa). This is ∼11% of the standard value for arterial $P_{CO_{2}}$ of 40 mmHg (5.3 kPa). For a three‐compartment model lung ventilated with air and with no significant shunt, it follows immediately that to generate an A‐a gradient of −11%, the mean percentage value for *˙V*_D,alv_/*˙V*_T_ must also be 11%. This prediction compares remarkably well with the overall mean value for *˙V*_D,alv_/*˙V*_T_ from this study of 11.5%.

The individual values for *˙V*_D,alv_/*˙V*_T_ were quite variable, with two standard deviations extending just beyond a value of 20%. Part of this will represent true variation between individuals, but individual estimates of *˙V*_D,alv_/*˙V*_T_ are also likely to be quite noisy. This is because *˙V*_D,alv_/*˙V*_T_ essentially depends on the A‐a gradient for $P_{CO_{2}}$, and this gradient is calculated by taking the difference between two substantially larger numbers (i.e., $P_{\mathrm{AC}O_{2}}$ and $P_{\mathrm{aC}O_{2}}$) that are both experimentally determined. Thus, a small percentage error in either of the large numbers will lead to a much larger percentage error in the difference.

Quantitatively, Farrow et al.’s (2023) values for both *˙V*_D,alv_/*˙V*_T_ (median 16.6 %) and *˙Q*_s_/*˙Q*_T_ (median 4.3%) were somewhat larger and more variable than ours. However, this may relate simply to differences between the two populations and between the two measurement techniques. Our participants were predominantly male, they were on average ∼10 years younger, they had lower average BMIs, they were probably fitter and while the smoking history was relatively similar between the two groups, fewer of our participants had either diabetes or hypertension. There may also have been differences in medical management of the participants between the two groups, and some of Farrow et al.’s (2023) participants may have been studied earlier following COVID‐19 than was the case for our participants. Qualitatively, however, the results were essentially the same between the two studies: Farrow et al. (2023) found *˙Q*_s_/*˙Q*_T_ increased with increasing COVID‐19 severity, but *˙V*_D,alv_/*˙V*_T_ did not change with increasing COVID‐19 severity. Biologically, many of the participants in the lowest COVID‐19 severity group were probably only suffering the effects of an upper respiratory tract infection. It is unlikely that this would exert a sustained increase on *˙V*_D,alv_/*˙V*_T_ and consequently it is hard to understand why any effect of prior COVID‐19 infection on *˙V*_D,alv_/*˙V*_T_ would not appear to be greater in the higher COVID‐19 severity groups.

The stark contrast in the conclusions drawn from the present study and those drawn by Farrow et al. relates less to differences in the underlying data from the two studies and more to Farrow et al.’s (2023) selection of a value of 10% as the upper limit of normal for *˙V*_D,alv_/*˙V*_T_. The citation they give for this limit is to a study of patients with asthma (Wagner et al., 1987), but within that study, a value of 0.6 is used for healthy volunteers as an upper limit of normal for logSDV. This is a parameter used in the multiple gas elimination technique (Roca & Wagner, 1994) to describe the *˙V*:*˙Q* distribution within the lung. While there is no exact correspondence between logSDV and alveolar deadspace, in practice, a value of 0.6 for logSDV corresponds to a contribution of *˙V*:*˙Q* mismatch to overall *˙V*_D,alv_/*˙V*_T_ of ∼5%. Farrow et al. (2023) chose to double this to 10% for their upper limit of normal. As a check on this limit, Farrow et al. (2023) studied a further seven (younger) healthy controls. *˙V*_D,alv_/*˙V*_T_ exceeded 10% in at least two (possibly three) of these seven participants. If we take the 95th centile as an upper limit of normal, then the probability of two or more of seven normal participants exceeding the threshold is <0.05. Thus, Farrow et al.’s (2023) study of seven young, healthy participants essentially refutes, rather than supports, the choice of an upper limit as low as 10%.

Clearly, upper limits of normal of 5% or even 10%, for *˙V*_D,alv_/*˙V*_T_, which have been determined using the multiple inert gas elimination technique, cannot fully explain the average values for *˙V*_D,alv_/*˙V*_T_ for CO_2_ of ∼11% determined in this study and predicted from the literature. Possible explanations include: (1) there is significant pure alveolar deadspace in normal individuals; (2) the multiple inert gas elimination technique has failed to identify significant regions of high *˙V*:*˙Q* ratio in normal individuals that would generate additional apparent alveolar deadspace; or (3) there are mechanisms other than *˙V*:*˙Q* mismatch that can generate apparent alveolar deadspace, at least in relation to CO_2_.

In relation to pure alveolar deadspace, both Permutt et al. (2009) and West et al. (1964) identified different zones within the lung based on the effects of gravity on the pulmonary circulation and this raised the possibility that an upper zone 1 could exist within the lung that is completely unperfused by blood from the right heart. At least in health, however, it seems unlikely that this zone actually exists because the systolic pulmonary arterial pressure should be sufficient to open the capillaries all the way to the apex of the lung.

With respect to the multiple inert gas elimination technique potentially missing high *˙V*:*˙Q* ratios within the lung, it has been suggested that the high‐solubility gases used in the technique, especially acetone, may exchange primarily within the airways rather than within the alveoli (Anderson et al., 2006) and in so doing could alter the measured excretion for these gases. Anderson and Hlastala (2010) explored this possibility through simulation and found an increase in ventilation at the higher *˙V*:*˙Q* ratios (demonstrated in their Figure 4). While these findings are of interest, it remains the case that they are simply the results from simulations based on a particular model of gas exchange in the airways, and they have not been verified experimentally.

The third possibility is that there are other mechanisms, possibly specific for CO_2_, that can contribute to apparent deadspace. For example, one possible cause is that, for a moderately soluble gas like CO_2_, there is a small, diffusion‐limited exchange between the gas and the walls of the conducting airways. If so, then the airway wall $P_{CO_{2}}$ would be lowered during inspiration and elevated during expiration, and that would result in the $P_{CO_{2}}$ of the gas at the mouth during expiration being slightly lower compared with when it left the alveoli. Thus, the A‐a gradient would be overestimated from measurements of $P_{CO_{2}}$ at the mouth.

Another possible mechanism is that there is some delay in the equilibration of CO_2_ with blood. Historically, until it was realised that carbonic anhydrase was present on the pulmonary endothelium, there was interest in the idea that the end‐capillary $P_{CO_{2}}$ would equilibrate with the alveolar $P_{CO_{2}}$, but after leaving the pulmonary capillary it would then rise as the slow conversion process from carbonic acid to CO_2_ and water continued to proceed in the plasma (which lacks carbonic anhydrase) downstream of the pulmonary capillary. While this explanation may no longer be tenable, it is not clear that others are impossible. The reactions affecting CO_2_ carriage by the blood are many and complex (Geers & Gros, 2000), a considerable fraction of the CO_2_ exchange is driven by the Haldane effect and so is dependent on the prior loading of haemoglobin with oxygen, this loading of haemoglobin by oxygen may be slower than previously thought (Richardson et al., 2020) and the kinetics associated with bicarbonate–chloride exchange through band III protein may also be significant (Hsu, 2018).

In summary, there are a number of possible explanations as to why data from both this study and that of Farrow et al. (2023) generate values for *˙V*_D,alv_/*˙V*_T_ for CO_2_ that are in excess of 10%. Some seem highly improbable, for example the presence of significant amounts of true alveolar deadspace in the lungs of normal individuals, but all are clearly speculative and whether any of them is correct remains to be determined.

## AUTHOR CONTRIBUTIONS

Dominic Sandhu, Grant A.D. Ritchie and Peter A. Robbins conceived and designed research; Dominic Sandhu and Snapper R.M. Magor‐Elliott analysed data; Dominic Sandhu and Peter A. Robbins interpreted results of experiments; Dominic Sandhu prepared figures; Peter A. Robbins drafted manuscript; Nick P. Talbot, Nayia Petousi, David A. Holdsworth, Alexander N. Bennett, Grant A.D. Ritchie and Peter A. Robbins edited and revised manuscript. Peter A. Robbins approved final version of manuscript. All authors have read and approved the final version of this manuscript and agree to be accountable for all aspects of the work in ensuring that questions related to the accuracy or integrity of any part of the work are appropriately investigated and resolved. All persons designated as authors qualify for authorship, and all those who qualify for authorship are listed.

## CONFLICT OF INTEREST

Oxford University Innovation, a wholly owned subsidiary of the University of Oxford, holds/has filed patents relating to the background IP for the technology. G.A.D.R. and P.A.R. have an interest in one or more patents. None of the other authors has any conflicts of interest, financial or otherwise, to disclose.

## Data Availability

Data will be made available upon reasonable request.

## References

[eph13931-bib-0001] Anderson, J. C. , & Hlastala, M. P. (2010). Impact of airway gas exchange on the multiple inert gas elimination technique: Theory. Annals of Biomedical Engineering, 38(3), 1017–1030. 20336837 10.1007/s10439-009-9884-x PMC3152310

[eph13931-bib-0002] Anderson, J. C. , Lamm, W. J. E. , & Hlastala, M. P. (2006). Measuring airway exchange of endogenous acetone using a single‐exhalation breathing maneuver. Journal of Applied Physiology, 100(3), 880–889. 16282431 10.1152/japplphysiol.00868.2005

[eph13931-bib-0003] Bjurstedt, H. , Hesser, C. M. , Liljestrand, C. , & Matell, G. (1962). Effects of posture on alveolar‐arterial CO_2_ and O_2_ differences and on alveolar deadspace in man. Acta Physiologica Scandinavica, 54(1), 65–82. 13869634 10.1111/j.1748-1716.1962.tb02329.x

[eph13931-bib-0004] Ciaffoni, L. , O'Neill, D. P. , Couper, J. H. , Ritchie, G. A. , Hancock, G. , & Robbins, P. A. (2016). In‐airway molecular flow sensing: A new technology for continuous, noninvasive monitoring of oxygen consumption in critical care. Science Advances, 2(8), e1600560. 27532048 10.1126/sciadv.1600560 PMC4980105

[eph13931-bib-0005] DuBois, A. B. , Britt, A. G. , & Fenn, W. O. (1952). Alveolar CO_2_ during the respiratory cycle. Journal of Applied Physiology, 4(7), 535–548. 14907569 10.1152/jappl.1952.4.7.535

[eph13931-bib-0006] Farrow, C. E. , Robles, R. A. , Prisk, G. K. , Harbut, P. , Malhotra, A. , Amis, T. C. , Wagner, P. D. , & Kairaitis, K. (2023). Increased intrapulmonary shunt and alveolar dead space post‐COVID‐19. Journal of Applied Physiology (1985), 135(5), 1012–1022. 10.1152/japplphysiol.00267.2023 PMC10911808 37767555

[eph13931-bib-0007] Geers, C. , & Gros, G. (2000). Carbon Dioxide transport and carbonic anhydrase in blood and muscle. Physiological Reviews, 80(2), 681–715. 10747205 10.1152/physrev.2000.80.2.681

[eph13931-bib-0008] Hsu, K. (2018). Exploring the potential roles of band 3 and aquaporin‐1 in blood CO2 transport–inspired by comparative studies of glycophorin B‐A‐B hybrid protein GP.Mur. Frontiers in Physiology, 9, 733. 29971013 10.3389/fphys.2018.00733 PMC6018491

[eph13931-bib-0009] Magor‐Elliott, S. R. M. , Alamoudi, A. , Chamley, R. R. , Xu, H. , Wellalagodage, T. , McDonald, R. P. , O'Brien, D. , Collins, J. , Coombs, B. , Winchester, J. , Sellon, E. , Xie, C. , Sandhu, D. , Fullerton, C. J. , Couper, J. H. , Smith, N. M. J. , Richmond, G. , Cassar, M. P. , Raman, B. , … Robbins, P. A. (2022). Altered lung physiology in two cohorts after COVID‐19 infection as assessed by computed cardiopulmonography. Journal of Applied Physiology, 133(5), 1175–1191. 36173325 10.1152/japplphysiol.00436.2022 PMC9639770

[eph13931-bib-0010] Marshall, J. C. , Murthy, S. , Diaz, J. , Adhikari, N. K. , Angus, D. C. , Arabi, Y. M. , Baillie, K. , Bauer, M. , Berry, S. , Blackwood, B. , Bonten, M. , Bozza, F. , Brunkhorst, F. , Cheng, A. , Clarke, M. , Dat, V. Q. , de Jong, M. , Denholm, J. , Derde, L. , … Zhang, J. (2020). A minimal common outcome measure set for COVID‐19 clinical research. The Lancet Infectious Diseases, 20(8), e192–e197. 32539990 10.1016/S1473-3099(20)30483-7 PMC7292605

[eph13931-bib-0011] Matell, G. (1963). Time‐courses of change in ventilation and arterial gas tensions in man induced by moderate exercise. Acta Physiologica Scandinavica, 58, 1–47.14013176

[eph13931-bib-0012] O'Neill, D. P. , & Robbins, P. A. (2017). A mechanistic physicochemical model of carbon dioxide transport in blood. Journal of Applied Physiology, 122(2), 283–295. 27881667 10.1152/japplphysiol.00318.2016 PMC5341128

[eph13931-bib-0013] O'Sullivan, O. , Barker‐Davies, R. , Chamley, R. , Sellon, E. , Jenkins, D. , Burley, R. , Holden, L. , Nicol, A. M. , Phillip, R. , Bennett, A. N. , Nicol, E. , & Holdsworth, D. A. (2021). Defence Medical Rehabilitation Centre (DMRC) COVID‐19 recovery service. British Medical Journal Military Health, 169, 271–276. 33547188 10.1136/bmjmilitary-2020-001681

[eph13931-bib-0014] Permutt, S. , Bromberger‐Barnea, B. , & Bane, H. N. (2009). Alveolar pressure, pulmonary venous pressure, and the vascular waterfall. Medicina Thoracalis, 19, 239–260. 10.1159/000192224 13942495

[eph13931-bib-0015] Richardson, S. L. , Hulikova, A. , Proven, M. , Hipkiss, R. , Akanni, M. , Roy, N. B. A. , & Swietach, P. (2020). Single‐cell O_2_ exchange imaging shows that cytoplasmic diffusion is a dominant barrier to efficient gas transport in red blood cells. Proceedings of the National Academy of Sciences, USA, 117(18), 10067–10078. 10.1073/pnas.1916641117 PMC7211990 32321831

[eph13931-bib-0016] Riley, R. L. , & Cournand, A. (1949). Ideal alveolar air and the analysis of ventilation‐perfusion relationships in the lungs. Journal of Applied Physiology, 1(12), 825–847. 18145478 10.1152/jappl.1949.1.12.825

[eph13931-bib-0017] Roca, J. , & Wagner, P. D. (1994). Contribution of multiple inert gas elimination technique to pulmonary medicine. 1. Principles and information content of the multiple inert gas elimination technique. Thorax, 49(8), 815–824. 8091330 10.1136/thx.49.8.815 PMC475132

[eph13931-bib-0018] Wagner, P. D. , Hedenstierna, G. , & Bylin, G. (1987). Ventilation‐perfusion inequality in chronic asthma. American Review of Respiratory Disease, 136(3), 605–612. 3631733 10.1164/ajrccm/136.3.605

[eph13931-bib-0019] Wagner, P. D. , Laravuso, R. B. , Uhl, R. R. , & West, J. B. (1974). Continuous distributions of ventilation‐perfusion ratios in normal subjects breathing air and 100 per cent O_2_ . Journal of Clinical Investigation, 54(1), 54–68. 4601004 10.1172/JCI107750 PMC301524

[eph13931-bib-0020] Wagner, P. D. , Malhotra, A. , & Prisk, G. K. (2022). Using pulmonary gas exchange to estimate shunt and deadspace in lung disease: Theoretical approach and practical basis. Journal of Applied Physiology, 132(4), 1104–1113. 35323050 10.1152/japplphysiol.00621.2021 PMC9022598

[eph13931-bib-0021] Wasserman, K. , Van Kessel, A. L. , & Burton, G. G. (1967). Interaction of physiological mechanisms during exercise. Journal of Applied Physiology, 22(1), 71–85. 6017656 10.1152/jappl.1967.22.1.71

[eph13931-bib-0022] West, J. B. , Dollery, C. T. , & Naimark, A. (1964). Distribution of blood flow in isolated lung; relation to vascular and alveolar pressures. Journal of Applied Physiology, 19(4), 713–724. 14195584 10.1152/jappl.1964.19.4.713

[eph13931-bib-0023] Whipp, B. J. , & Wasserman, K. (1969). Alveolar‐arterial gas tension differences during graded exercise. Journal of Applied Physiology, 27(3), 361–365. 5804133 10.1152/jappl.1969.27.3.361

